# GO-guided direct growth of highly oriented metal–organic framework nanosheet membranes for H_2_/CO_2_ separation†
†Electronic supplementary information (ESI) available. See DOI: 10.1039/c7sc04815g


**DOI:** 10.1039/c7sc04815g

**Published:** 2018-04-02

**Authors:** Yujia Li, Haiou Liu, Huanting Wang, Jieshan Qiu, Xiongfu Zhang

**Affiliations:** a State Key Laboratory of Fine Chemicals , School of Chemical Engineering , Dalian University of Technology , Dalian , 116024 , China . Email: xfzhang@dlut.edu.cn; b Department of Chemical Engineering , Monash University , Clayton , Victoria 3800 , Australia

## Abstract

Highly oriented, ultrathin metal–organic framework (MOF) membranes are attractive for practical separation applications, but the scalable preparation of such membranes especially on standard tubular supports remains a huge challenge. Here we report a novel bottom-up strategy for directly growing a highly oriented Zn_2_(bIm)_4_ (bIm = benzimidazole) ZIF nanosheet tubular membrane, based on graphene oxide (GO) guided self-conversion of ZnO nanoparticles (NPs). Through our approach, a thin layer of ZnO NPs confined between a substrate and a GO ultrathin layer self-converts into a highly oriented Zn_2_(bIm)_4_ nanosheet membrane. The resulting membrane with a thickness of around 200 nm demonstrates excellent H_2_/CO_2_ gas separation performance with a H_2_ performance of 1.4 × 10^–7^ mol m^–2^ s^–1^ Pa^–1^ and an ideal separation selectivity of about 106. The method can be easily scaled up and extended to the synthesis of other types of Zn-based MOF nanosheet membranes. Importantly, our strategy is particularly suitable for the large-scale fabrication of tubular MOF membranes that has not been possible through other methods.

## Abstract 2

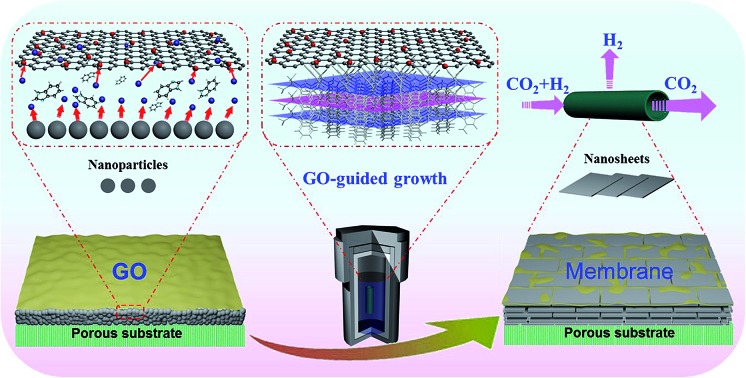
A highly oriented 2D nanosheet metal–organic framework membrane is fabricated by a direct growth strategy.

## 1. Introduction

Two-dimensional (2D) membranes fabricated with 2D materials such as graphene, graphene oxide (GO), zeolites, and metal–organic frameworks (MOFs) have attracted intense research interest due to their unique physical and chemical properties originating from their highly oriented pores, ultrathin thickness and 2D morphology.^1^–^4^ Since the pioneering work on fabricating GO membranes using GO flakes as building blocks, considerable effort has been devoted to the preparation of different kinds of 2D membranes.^5^–^8^

In recent years, as a new member of the 2D family, 2D MOF nanosheets are receiving increasing attention because of their large internal surface areas, uniform channels, (sub)nanometer-sized cavities, thermal stability, and chemical tailorability.^9^–^12^ In particular, 2D MOF nanosheets possess many accessible active sites on the surface, which could be significant for gas storage, sensors, chemical catalysis, adsorption and small-molecule separations.^13^–^17^ Two strategies are proposed for the preparation of 2D nanosheets, *i.e.*, top-down and bottom-up methods.^18^–^23^ The former involves the delamination of bulk materials, while the latter is direct synthesis of 2D nanosheets. However, until now, the top-down method, including first exfoliation and then coating the obtained thin nanosheets on a porous substrate, has been the main strategy to fabricate 2D MOF membranes. Yang's group^19^ for the first time used this top-down strategy to prepare an ultrathin 2D ZIF membrane supported on a porous disc, achieving super-selective molecular sieving properties for H_2_/CO_2_ separation. On the other hand, Gascon *et al.*^23^ synthesized ultrathin CuBDC (BDC = 1,4-benzenedicarboxylate) nanosheets by the bottom-up method to prepare a mixed matrix membrane (MMM) with superior CO_2_/CH_4_ separation performance.

In fact, the top-down preparation method inevitably involves the exfoliation step which usually causes some fragmentation and morphological damage of the detached sheets.^23^,^24^ Moreover, it is also rather complex, arduous, costly and time-consuming. Furthermore, the deposition or coating step of exfoliated nanosheets on a porous substrate is apt to cause overlap and crumpling of the nanosheets, thus leading to the pore interleaving and low orientation of the achieved nanosheet membrane that gives a low separation performance. Therefore, it is difficult to apply the top-down preparation method to large-scale fabrication of nanosheet membranes on tubular substrates for industrial applications. In principle, the direct synthesis of 2D MOF membranes by the bottom-up method can overcome these shortcomings mentioned above and be especially suitable for the large-scale preparation for practical applications. But it still faces a big challenge. To the best of our knowledge, until now, there has been no report on the fabrication of a crystalline nanosheet membrane on a porous tubular substrate by direct growth.

**Fig. 1:**
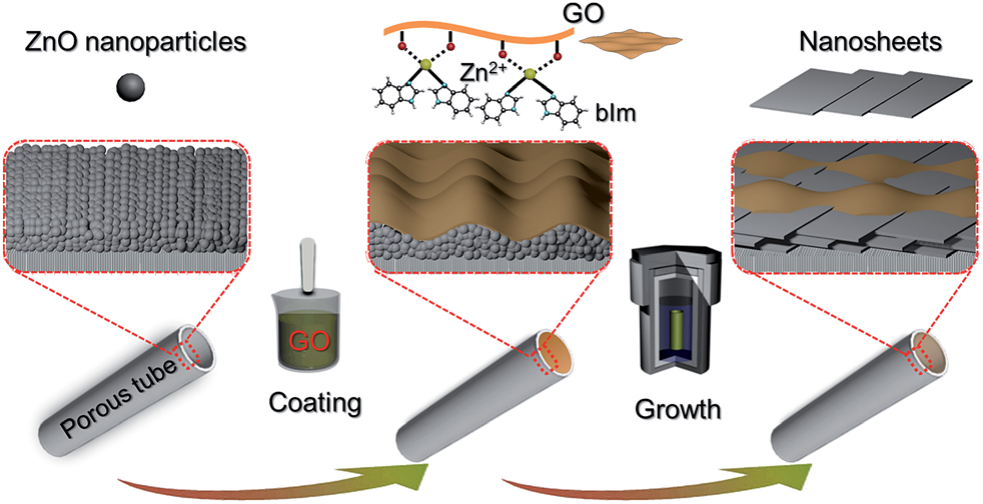
Scheme depicting the preparation procedure of highly oriented Zn_2_(bIm)_4_ nanosheet membranes by ZnO self-conversion growth in a GO confined space.

In our previous study, a 2D ZIF nanosheet membrane was achieved by a self-conversion growth of ZnO nanoparticles (NPs).^25^ However, the orientation of the achieved nanosheet membrane is still unsatisfactory, which seriously influences the microstructure and separation performance of the membrane. Moreover, the operation process is rather complicated and the reproducibility of membrane preparation is poor. Herein, we, for the first time, present a bottom-up methodology for fabricating a highly oriented MOF membrane consisting of 2D nanosheets supported on a porous *tubular* substrate for H_2_/CO_2_ separation by the GO-guided self-conversion of ZnO NPs. As shown in Fig. 1, this artful strategy combines two important concepts as follows: firstly, a thin layer of metal oxide NPs coated on a porous tubular substrate is employed as seeds to produce 2D MOF nanosheets and further form a continuous membrane with 2D nanosheet architecture by self-conversion of the thin layer of metal oxide NPs in a ligand solution. The metal oxide NP layer on the porous substrate by coating and calcination steps plays a multifunctional role: (1) acting as the metal source and providing nucleation sites for the growth of the MOF nanosheet membrane, which is beneficial to forming a continuous and uniform membrane; (2) facilitating anchoring between the membrane and porous substrate for achieving a stable nanosheet membrane; (3) adjusting the membrane thickness which mainly relies on the thickness of the metal oxide NP layer. Secondly, an ultrathin layer of graphene oxide (GO) placed on the top of the thin layer of metal oxide NPs is employed to confine and control the orientation growth of the nanosheet membrane, producing a highly oriented nanosheet MOF membrane. The ultrathin layer of GO also plays two key roles in the formation of the nanosheet membrane. On the one hand, the GO layer placed on the top of the metal oxide NP layer guides the growth direction of nanosheets under it and avoids the random growth of nanosheets, thus ensuring the formation of a highly oriented nanosheet membrane. It is well known that GO, as a two-dimensional graphene-based nanomaterial, possesses abundant polar oxygen functional groups and aromatic sp^2^ domains, and thus it can act as structural nodes and participate in bonding interactions with MOFs.^26^,^27^ On the other hand, GO may also cover and remedy some defects of the nanosheet membrane possibly produced during the assembly of the nanosheet membrane, therefore favoring the formation of a perfect nanosheet membrane. Our work provides a simple and scalable bottom-up method for fabricating a highly oriented nanosheet membrane supported on a porous tubular substrate.

We chose Zn_2_(bIm)_4_ (Zn_2_(benzimidazole)_4_) as an example to demonstrate our GO-guided metal oxide self-conversion strategy, as the Zn_2_(bIm)_4_ units are considered as subunits of the 2D structure, in which the apices are occupied by Zn atoms and the sides are formed by benzimidazole ligands. Its aperture size, a four-membered ring, is no more than 0.21 nm. Thus, the 2D Zn_2_(bIm)_4_ membrane can demonstrate excellent performance in separating H_2_/CO_2_ mixtures and have potential applications in some small-molecule gas separations.^19^

## 2. Experimental

### 2.1 Materials and reagents

The commercial porous α-alumina tubes with 4 mm OD (outer diameter), 3 mm ID (inner diameter) and an average pore size of 100 nm as substrates were purchased from Hyflux Ltd. Co. The tubes were cut into 60 mm lengths and washed sequentially with distilled water and ethanol several times. The tubes were then dried at 100 °C for 5 h before calcination at 550 °C for 6 h.

The chemicals used in the membrane preparation include zinc nitrate hexahydrate (≥99.0%), zinc acetate (98.0%), ethylene glycol monomethyl ether (C_3_H_8_O_2_, EGME, 99.0%), toluene (≥99 wt%), ammonium hydroxide (NH_3_, 28–30% aqueous solution), *N*,*N*-dimethylformamide (DMF, ≥99 wt%), monoethanolamine (C_2_H_7_NO, MEA, 99.0%) and anhydrous methanol (≥99.5%) purchased from Sinopharm Chemical Reagent Co., Ltd. Benzimidazole (bIm, ≥99 wt%), phthalic acid (BDC, ≥99 wt%) and 1,3,5-benzenetricarboxylic acid (BTC, ≥99 wt%) were supplied by Sigma-Aldrich Chemical. Co. Ltd. Zinc oxide nanoparticles (cal. 40 ± 10 nm) were purchased from Beijing Nachen S&T Ltd.

### 2.2 Preparation of a thin layer of ZnO NPs

According to previous ref. 28 and 29, a sol containing about 14.3 wt% zinc was prepared and used to coat the inner wall of the porous tube by using a dip-coating technique. The outer surface of the tube was first wrapped with Teflon tape and then dip-coated for the desired time, followed by drying at 100 °C for 1 h. This procedure was repeated twice to obtain a ZnO layer with around 50 ± 5 nm thickness on the tube. The tube was then calcined at 400 °C in air for 2 h.

### 2.3 Preparation of an ultrathin layer of GO on the ZnO layer coated tube

The GO suspension was prepared from natural graphite powder using the modified Hummers method as reported in the literature.^30^,^31^ The suspension was further diluted with methanol, followed by sonication for 2 h to obtain a final GO suspension of 0.25 mg ml^–1^ in which the volume ratio of water to methanol was about 1 : 4. The tube with the ZnO NP layer was coated with the above GO solution using the dip-coating technique, followed by drying at 100 °C for 4 h to achieve a dual-layer ZnO NPs@GO supported on the porous tube with the GO layer of 20 ± 5 nm thickness.

### 2.4 Growth of a highly oriented nanosheet membrane

According to our previous studies,^28^,^29^ a modified synthesis solution with benzimidazole (bIm) : NH_4_OH : MeOH : toluene (PhMe) with a molar composition of 1 : 1 : 45 : 45 was first prepared for the formation of ZIF nanosheets. Then, the tube with both ZnO NP and GO layers was placed in the synthesis solution at 100 °C for the desired reaction time. After the synthesis, the membrane was taken out, immersed in methanol for 2 days and then thoroughly rinsed with methanol. Finally, the membrane was dried at room temperature overnight and then stored in a desiccator for later use.

### 2.5 Materials characterization and gas permeation

X-ray diffraction (XRD) measurements were performed on a D/max-2400 X-ray diffractometer using Cu Kα radiation in the range of 3–60° operating at 20 kV/100 mA. Scanning electron microscopy (SEM) images were obtained with a NOVA NANOSEM 450 SEM (FEI Company) and a Tecnai F30 transmission electron microscope (TEM). The settings of the SEM were as follows: high voltage (HV) 5–15 kV, working distance (WD) 5–10 mm and spot size 3.0. Fourier transform infrared (FTIR) spectra (4000–400 cm^–1^) were recorded on a Bruker Equinox 55 spectrometer in KBr plates. Thermal gravimetric analysis (TGA) in a DTU-2 was done in flowing air from 25 °C to 700 °C at 10 °C min^–1^. AFM micrographs were recorded with a Veeco Multimode Nanoscope 3A microscope operating in tapping mode. The MOF samples were applied to a previously annealed mica wafer substrate. Before microscopy inspection, a couple of drops from a suspension of nanosheets in methanol were applied and allowed to dry over the mica substrate. The surface elemental composition was obtained by X-ray photoelectron spectroscopy (XPS, Physical Electronics PHI 5000) using a monochromatic Al X-ray source (1486.6 eV).

Gas permeances of the highly oriented nanosheet membranes achieved were measured for single gases including H_2_ (0.29 nm), CO_2_ (0.33 nm), N_2_ (0.36 nm) and CH_4_ (0.38 nm), while the separation of binary gas mixtures was investigated for H_2_/CO_2_, H_2_/N_2_ and H_2_/CH_4_. The experimental setup is shown in Fig. S1 of the ESI.† Single gas permeation tests were conducted by pressurizing the feed stream, while keeping the permeate pressure at 1 atmosphere. The gas permeances at transmembrane pressures of 0.06 MPa to 0.12 MPa were measured. The membrane was purged to remove entrained gases prior to each experimental run. The permeance, *P*_*i*_, is defined by following eqn (1): where *N*_*i*_ is the permeation rate of component *i* (mol s^–1^), Δ*P*_*i*_ is the trans-membrane pressure difference of *i* component (Pa), and *A* is the membrane area (m^2^). *F*_*i*_ is the flux of component *i* (mol m^–2^ s^–1^). The ideal separation factor is calculated as the ratio of permeance *P*_*i*_ and *P*_*j*_ in eqn (2).
1

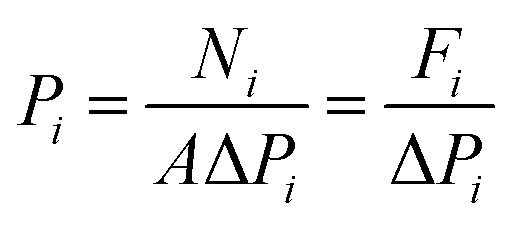



2

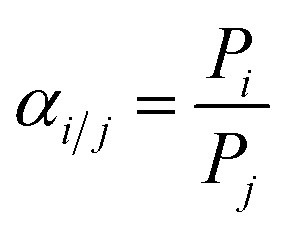




The separation properties were investigated for equimolar binary gas mixtures (H_2_/CO_2_, H_2_/N_2_ and H_2_/CH_4_). The feed gas was constant at a 1 : 1 volume ratio. The total pressure on each side of the membrane was atmospheric. Nitrogen was used as the sweep gas on the permeate side for H_2_/CO_2_ and H_2_/CH_4_ mixtures, while methane was used for the H_2_/N_2_ mixture. The gas composition was analyzed using an online gas chromatograph (GC7890T). The separation selectivity *α*_*i*/*j*_ for the binary gas mixtures was defined as the following eqn (3): where *X*_*i*_ and *X*_*j*_ are the molar fractions of components *i* and *j* in the retentate stream which is similar to the feed component, while, *Y*_*i*_ and *Y*_*j*_ are the molar ratios of components *i* and *j* on the permeate side.
3

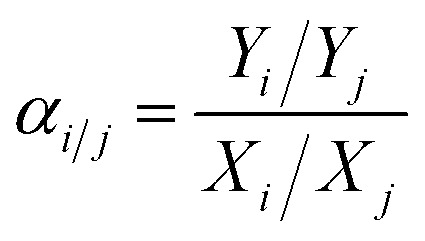




## 3. Results and discussion

### 3.1 Formation and characterization of oriented nanosheet membranes

Generally, to achieve an oriented MOF nanosheet membrane on a porous substrate in the synthesis solution by growth, two key points need to be considered. One is that the synthesis solution can generate MOF nanosheets, which ensures that the grown membrane is composed of nanosheets. The other is that the nanosheets have to grow in the same direction into a continuous membrane. Both the aspects lead to an oriented nanosheet membrane.

**Fig. 2:**
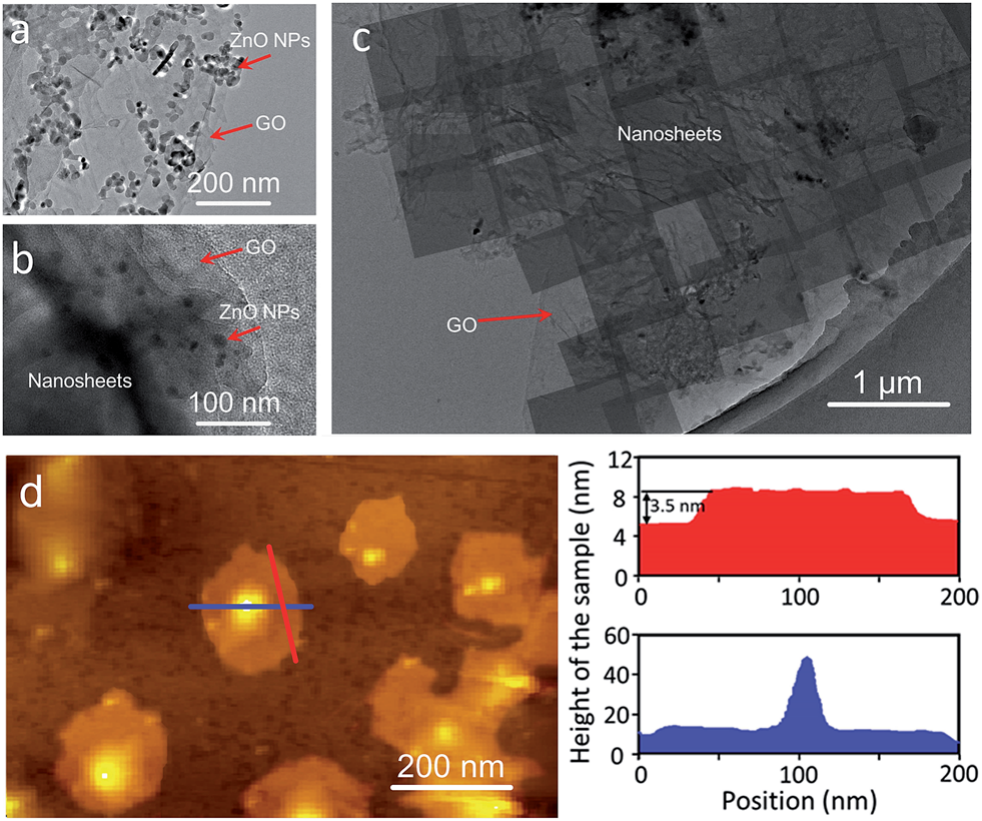
TEM images of the ZnO@GO nanocomposite (a) and the samples (b and c) achieved by self-conversion of ZnO NPs@GO in the synthesis solution after reaction for 1 h and 5 h, respectively. (d) Atomic-force micrograph (with corresponding height profiles), height image and height profiles for the nanosheets cast on a silicon wafer. The height profiles of the nanosheets are extracted along the red and blue lines.

We first investigated the conversion of ZnO NPs@GO composite particles obtained by the coating of ZnO NPs with GO in the benzimidazole ligand solution with different treatment times by using bright-field transmission electron microscopy (BR-TEM). As shown in Fig. 2a–c, the diameter of the ZnO NPs coated with GO is around 40 ± 10 nm before the reaction (Fig. 2a). In the early stage of the reaction (1 h), some nanosheet precursors are formed around the surfaces of the ZnO NPs (Fig. 2b). As the reaction proceeds, the nanosheets gradually grow larger, while the ZnO NPs become smaller, until the NPs are completely converted to the ZIF nanosheets (Fig. 2c). These results suggest that the nanosheets were mainly formed through the localized self-conversion of the ZnO NPs, rather than by dissolution of ZnO and following crystallization.^32^–^34^ The GO coated around ZnO NPs can provide anchoring sites for the oriented growth of nanosheets. This localized conversion phenomenon is important for further membrane fabrication since it allows the nanosheets to grow along GO on the substrate rather than in solution. This synthesis solution is also suitable for the self-conversion of the ZnO NPs@GO composite into ZIF nanosheets. The atomic force microscopy (AFM) image (Fig. 2d) shows that the resulting nanosheets cast on a silicon wafer have square lateral dimensions of around 100 nm and thicknesses in the range of 3–4 nm which result in nanosheets with aspect ratio exceeding 25.

**Fig. 3:**
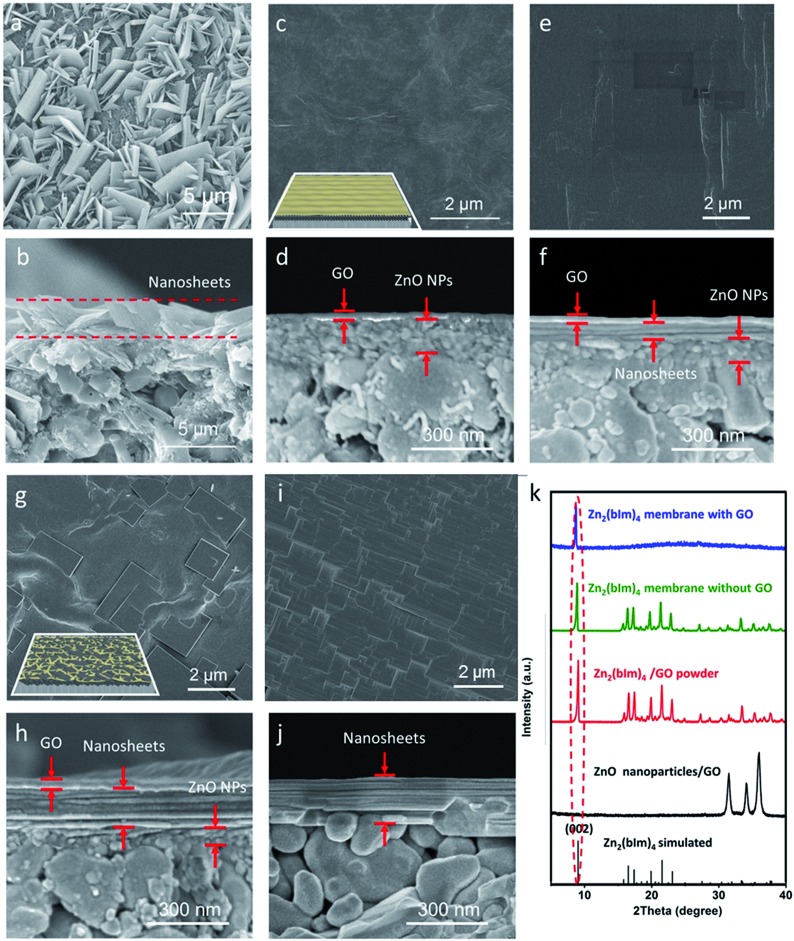
(a and b) SEM images of the samples obtained by self-conversion of ZnO NPs without the GO layer for the reaction for 5 h; (c–j) SEM images of the ZnO NPs@GO dual layer on the porous substrate (c and d) and the samples obtained by self-conversion of the ZnO NPs@GO layer for different reaction times of 1 h, 5 h and 9 h, respectively (e–j); (k) XRD patterns of the Zn_2_(bIm)_4_ membranes grown with GO and without GO, respectively, ZnO NPs@GO powder, Zn_2_(bIm)_4_/GO powder and the simulated pattern of Zn_2_(bIm)_4_ (ref. no. 675375, Cambridge Crystallographic Data Centre). Among them, (a, c, e, g and i) the surfaces; (b, d, f, h and j) the cross-sections; inset: the layered structure.

For comparison, a thin layer of ZnO NPs coated on a porous substrate was directly converted to grow a ZIF nanosheet membrane in the ligand synthesis solution for 5 h without using any GO. As shown in Fig. 3a, b and S5,† a layer of random-directed ZIF membrane with a thickness of around 2 μm consisting of nanosheets was formed. All the nanosheets were randomly deposited on the substrate surface, though they were partly intergrown. The XRD pattern (Fig. 3k) clearly shows that there are all peaks for the Zn_2_(bIm)_4_ structure, indicating that this membrane is not oriented. This mainly results from the reason that the nucleation and self-conversion growth of ZnO NPs is able to take place in any direction on the substrate surface in the absence of orientational control, thus resulting in the formation of a layer of randomly oriented multiple nanosheets. Therefore, to date, few continuous 2D nanosheet MOF membranes have been successfully prepared using the direct growth method.

Here, to obtain an oriented ZIF membrane, a thin layer of GO acting as orientational control was employed to coat the top of the ZnO NP layer supported on the porous substrate to form a sandwich-like structure in which the ZnO NP layer is sandwiched between the GO and surface of the substrate, realizing an oriented growth of a nanosheet membrane. As shown in Fig. 3c–j, the self-conversion growth process of the sandwich ZnO NPs@GO layer supported on the porous tube in the ligand solution with time was traced. It is clear that there was a two-layer structure consisting of the GO top layer of 20 ± 2 nm and ZnO NP bottom layer of 200 ± 10 nm on the substrate surface without any treatment (Fig. 3c and d). After a short synthesis time (1 h) in the ligand solution, the GO layer still remained intact, but the ZnO NP layer under the GO layer partly changed (Fig. 3e and f). An ultrathin layer of nanosheet membrane of around 50 ± 10 nm was formed between the GO layer and the top layer of the ZnO NPs, indicating that the top of the ZnO NP layer first began to self-convert into a nanosheet membrane under the limit of the GO layer. When the reaction time was increased to 5 h, the nanosheet membrane gradually became thicker by layer by layer stacking, while the ZnO NP layer was inversely thinner (Fig. 3g and h). Moreover, from the surface view, GO is partly interweaved in the nanosheets to form a ZIF nanosheets/GO interlaced structure in which GO favors remedying some defects generated from the nanosheet membrane assembly and strengthening the membrane stability. When the reaction time was further extended to 9 h, the ZnO NP layer was completely converted to a thick nanosheet membrane of about 200 nm thickness with a multilayer structure (Fig. 3i and j). The GO layer could hardly be observed at that time possibly because it was covered by the nanosheets or changed into fragments *via* some reactions in the alkaline synthesis solution.^35^ Most importantly, the X-ray diffraction pattern of the nanosheet membrane shows that there is only one reflection which can be indexed to the (002) crystallographic plane of the Zn_2_(bIm)_4_ of a highly oriented nanosheet membrane formed by self-conversion of ZnO NPs in a confined space of GO.

**Fig. 4:**
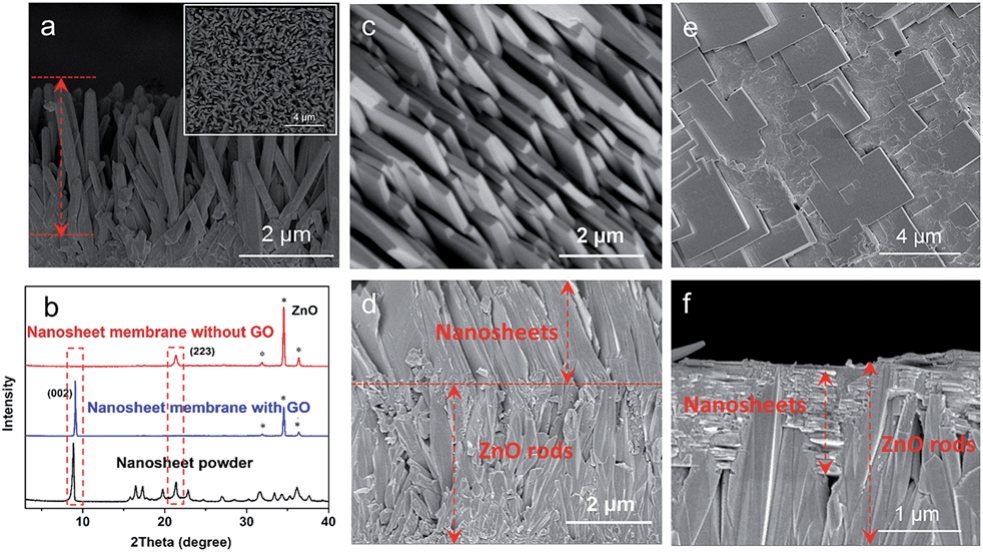
SEM images of the cross-section of the ZnO nanorod layer grown on the porous substrate (inset: the surface of ZnO nanorods) (a), the nanosheet membranes grown from the ZnO nanorods coated without the GO layer (c and d) and with the GO layer (e and f); (b) XRD patterns of ZIF nanosheet powders, the nanosheet membranes achieved on the ZnO nanorods coated with GO and without GO, respectively. (c and e) Surface; (d and f) cross-section.

To further verify the guiding function of the GO layer on the top of the ZnO NP layer, a layer of ZnO nanorods (NRs), instead of ZnO NPs, was grown on the porous substrate, followed by a thin layer of GO *via* coating to form a ZnO NRs@GO layer supported on the substrate for growing a nanosheet membrane under the same synthesis conditions. As shown in Fig. 4, after the growth in the synthesis solution on a layer of ZnO NRs of around 4 μm grown on the porous substrate (Fig. 4a), completely different results were generated on the ZnO NRs with the GO layer and without the GO layer, respectively. Without any GO layer, a continuous nanosheet ZIF membrane of around 2.3 μm can also be achieved (Fig. 4c and d), however, all the single-layer nanosheets vertically stand face to face on the top of the ZnO NR layer with the growth direction perpendicular to the surface of the porous substrate to form an oriented membrane. XRD data of the membrane also show only one reflection, which can be indexed to the (223) crystallographic plane of the ZIF structure (Fig. 4b). Surprisingly, with a thin layer of GO on the top of the ZnO NR layer, the oriented growth direction of nanosheets is completely changed. They grow horizontally along undersurface of the GO layer and between the ZnO NRs (Fig. 4f). The surface of the membrane is composed of nanosheets (Fig. 4e) that are similar to those achieved on the above ZnO NPs (Fig. 3g and i). The XRD pattern of the membrane only gives one reflection indexed to the (002), thus demonstrating a highly oriented growth with GO guidance. These differences may result from different growth mechanisms of the nanosheet membranes with the GO layer or without the GO layer as described in the following section. It is evident from the above results that the GO layer is stable and can play a confining role and guides the oriented growth of the ZIF nanosheet membrane, though it is possibly reduced partly under these synthesis conditions. As shown in the IR spectra of Fig. S7,† after the treatment with the same synthesis conditions, the three characteristic peaks of GO decreased slightly compared with GO before the reaction. They are still observed obviously, indicating that GO is not completely reduced to graphene during the reaction.

According to the crystallographic data calculated from XRD patterns, the 3D crystalline structure of the ZIF nanosheet was achieved on the surface modified with ZnO NPs [ref. no. 675375, Cambridge Crystallographic Data Centre (CCDC)].

The pore direction of the Zn_2_(bIm)_4_ consisting of 2D layers is in agreement with that of the substrate. The aperture size of the ZIF is estimated to be 0.21 nm as shown in Fig. S8.† The plane is perpendicular to the pore openings of the substrate, which greatly favors gas permeation.

### 3.2 Role of the GO layer and growth mechanism of oriented nanosheet membranes

**Fig. 5:**
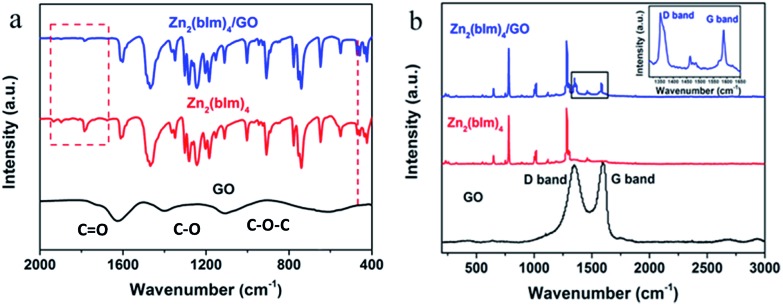
(a) FTIR spectra of GO, Zn_2_(bIm)_4_ nanosheets and Zn_2_(bIm)_4_ membrane-9; (b) Raman spectra of GO, Zn_2_(bIm)_4_ nanosheets and Zn_2_(bIm)_4_ membrane-9.

The above discussion indicates that the GO layer plays a key role in guiding the oriented growth of the Zn_2_(bIm)_4_ nanosheet membrane using this bottom-up method. To investigate the role of the GO layer during the preparation of the membrane and further clarify the formation mechanism of the highly oriented membrane, a series of characterizations of the samples were carried out by using FTIR and XPS techniques. The FTIR spectra of Zn_2_(bIm)_4_ nanosheets, GO, and Zn_2_(bIm)_4_ grown for 9 h (M-9) achieved after the reaction for 9 h are shown in Fig. 5a. In the spectra of GO, the characteristic peaks of GO appearing at 1680, 1400, and 1100 cm^–1^, representing C=O in carbonyl, and C–O and C–O–C in the epoxy group, respectively, are obviously present.^36^,^37^ Both Zn_2_(bIm)_4_ nanosheets and Zn_2_(bIm)_4_ M-9 have similar FTIR spectra. However, in the spectra of the Zn_2_(bIm)_4_ M-9, the intensities of the peaks at 1930 cm^–1^, 1900 cm^–1^ and 1780 cm^–1^ decreased and that of the peak at 470 cm^–1^ increased, showing the formation of a chemical covalent bond of Zn–O between GO and Zn_2_(bIm)_4_ because the carboxyl groups and epoxy groups of GO can interact with Zn^2+^ to form Zn–O bonds.^37^ The Raman spectra can further provide complementary structural information as shown in Fig. 5b. In the spectrum of GO, there are two peaks: the G band (1580 cm^–1^) related to the coplanar vibration of sp^2^ bonded carbon atoms and the D band (1340 cm^–1^). The D (associated with the order/disorder of the system) and G (an indicator of the stacking structure) bands of the Raman spectra are the dominant vibrational modes observed in graphitic structures.^37^,^38^ The intensity ratio of the bands (*I*_D_/*I*_G_) is often used as a means of determining the number of layers and their overall stacking behavior in a graphene sample. A high *I*_D_/*I*_G_ ratio indicates a high degree of exfoliation/disorder. It is found that the *I*_D_/*I*_G_ value of the Zn_2_(bIm)_4_ membrane-9 increased from 0.96 to 1.28 compared with GO. This should be ascribed to the presence of abundant covalent bonds of Zn–O formed by the interaction of C–O and C=O of GO with ZnO NPs.

From the above analysis of FTIR and Raman spectra, it is obvious that the GO on the top of the ZnO NP layer can interact with ZnO to produce Zn–O covalent bonds in the ligand solution, which favors the nucleation and oriented growth of the ZIF membrane.

**Fig. 6:**
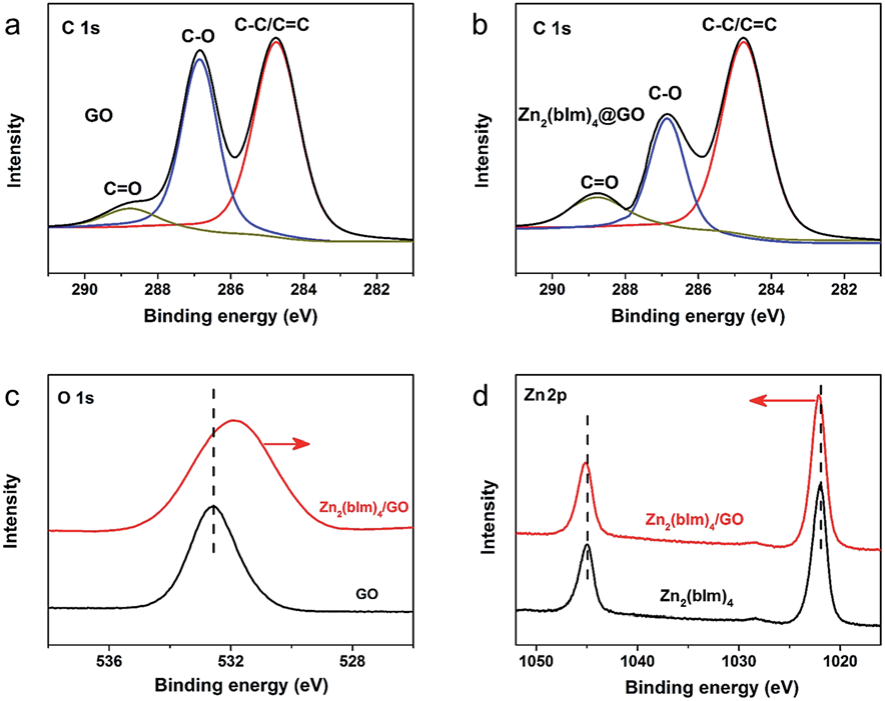
XPS spectra of different samples: (a) C 1s region of GO; (b) C 1s region of the Zn_2_(bIm)_4_/GO nanosheet composite; (c) high-resolution O 1s spectra of GO and the Zn_2_(bIm)_4_/GO nanosheet composite; (d) Zn 2p spectra of Zn_2_(bIm)_4_ and the Zn_2_(bIm)_4_/GO nanosheet composite.

**Fig. 7:**
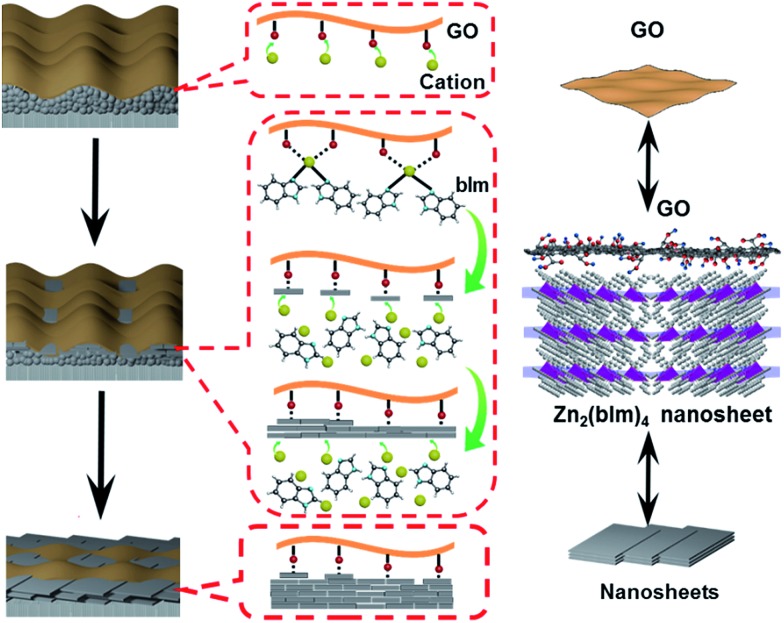
Formation mechanism of a highly oriented nanosheet membrane with the aid of GO on a porous alumina substrate.

To further confirm the reaction between the functional groups of GO and ZnO NPs, XPS spectra for the samples were recorded as shown in Fig. 6. Fig. 6a and b show the C 1s deconvolution spectra of GO and the Zn_2_(bIm)_4_/GO composite. The C 1s peaks of GO consist of three components arising from C–C/C=C, C–O and C=O.^39^–^41^ Compared with GO, the C–C/C=C peaks of the Zn_2_(bIm)_4_/GO composite become predominant, while the peaks of carbon atoms bonded to oxygen (C–O) are weakened remarkably, implying the conversion of the C–O group into the Zn–O bond by interaction with ZnO in the solution. From Fig. 6c and d, for the Zn_2_(bIm)_4_/GO, the peak in the O 1s spectrum displays a skewing to low binding energy, yet the binding energies of Zn-based bonds of the Zn_2_(bIm)_4_/GO composite are larger than those of Zn_2_(bIm)_4_. This should be ascribed to the fact that the electron cloud of bonds is shifted from Zn to O atoms, thus confirming that a Zn–O coordination bond between the O of the GO and Zn in Zn_2_(bIm)_4_ is formed.^42^ Therefore, this should also favor the nucleation of Zn_2_(bIm)_4_ nanosheets and the formation of the membrane because GO with abundant oxygen groups can play a seed role. Based on the above characterizations and analyses, a possible formation mechanism for the highly oriented Zn-based MOF nanosheet membrane is proposed as indicated in Fig. 7. Generally, without any GO layer, the ZnO NP layer coated on a porous substrate can act as seeds for the formation of a corresponding Zn-MOF membrane, but the achieved membrane is usually composed of neither nanosheets nor oriented structure.^28^,^29^,^43^ With a thin layer of GO coated on the top of the ZnO NP layer, the nucleation of Zn_2_(bIm)_4_ nanosheets should start from the interface between GO and the top of the ZnO NP layer because the C–O/C=O groups of the GO surface easily react with Zn^2+^ from ZnO NPs in the ligand solution. The ZnO NPs coming into contact with GO will preferentially react with the ligands to generate Zn_2_(bIm)_4_ nanosheets. Then, the formed nanosheets act as a seed-like template to induce the conversion of the bottom ZnO NPs into nanosheets downward under the GO restriction. With the reaction time, the nanosheets are gradually produced and then extended in a horizontally oriented direction along the surface of the GO sheet layer to form a continuous nanosheet membrane. Moreover, under the restricted state of the GO layer, the nanosheets in a parallel direction can only grow down toward the ZnO NP layer and stack into a multilayered nanosheet Zn_2_(bIm)_4_/GO membrane. Simultaneously the ZnO NPs are also consumed gradually and are finally used up with the increase of the nanosheet membrane thickness. Thus, in this method, the thickness of the ZnO NP layer determines that of the corresponding nanosheet membrane, while the GO layer on the top of the ZnO NP layer plays a key role in restricting and guiding the growth direction of the nanosheet membrane. Both these aspects lead to a highly oriented nanosheet MOF membrane. During the assembly of the nanosheet membrane, the part of the GO layer acting as nanosheets is possibly interweaved with ZIF nanosheets to form a ZIF nanosheet membrane interlaced with GO. Therefore, GO may remedy some defects possibly generated from the nanosheet membrane, thus leading to a high-performance membrane compared with the membrane obtained without any GO layer.

### 3.3 Gas permeation performance of the oriented nanosheet membranes

**Fig. 8:**
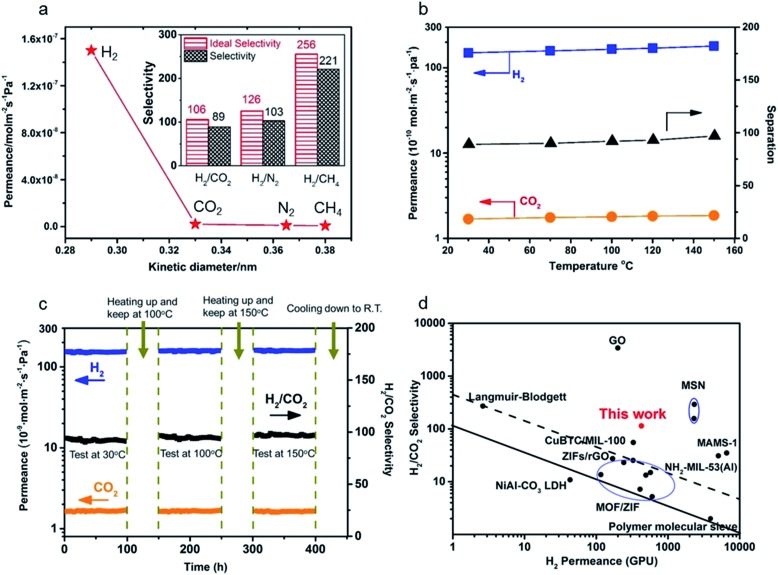
(a) Single gas permeances of the M-9 membrane (inset: ideal separation factors for single gases and separation selectivities for binary gas mixtures for H_2_ over CO_2_, N_2_ and CH_4_); (b) permeances of binary gas mixtures as a function of temperature difference; (c) long-term operating stability of the M-9 membrane for the separation of an equimolar H_2_/CO_2_ mixture in the range of temperatures from 30 to 150 °C at 0.1 MPa; (d) comparison of our M-9 membrane with the reported molecular sieve membranes for the separation of H_2_/CO_2_ mixtures.^14^,^19^,^23^,^28^,^42^,^47^–^61^ The black solid line represents the 2008 upper bound of polymeric membranes for H_2_/CO_2_. The black dashed line represents the 2010 upper bound of microporous inorganic membranes for the separation of H_2_/CO_2_ mixtures.

To evaluate the entire quality of the oriented nanosheet tubular membrane achieved after the solvothermal growth of 9 h (denoted as M-9), gas permeation performances with single and binary mixtures were measured, respectively. Fig. 8a shows the permeances of single and binary mixtures through the M-9 membrane as a function of the kinetic diameter of the permeating molecules at 30 °C. The gas permeances (H_2_, N_2_, CO_2_ and CH_4_) through the membrane decreased rapidly with increasing the kinetic diameter of gas molecules. The smallest H_2_ gas showed the highest permeance among these gases, while the permeances of larger gases were far lower than that of H_2_. This indicated that the as-prepared membrane exhibited excellent molecular sieve performance for H_2_ over other gases. The ideal selectivities were as high as 106, 126 and 256 for H_2_/CO_2_, H_2_/N_2_ and H_2_/CH_4_, respectively, which are far beyond their corresponding Knudsen selectivities (4.7, 3.7 and 2.8, respectively). Moreover, the molecular sieve performance of the M-9 membrane was confirmed by the separation of equimolar mixtures of H_2_ and CO_2_, N_2_ and CH_4_, and the separation selectivities for H_2_/CO_2_, H_2_/N_2_ and H_2_/CH_4_ at 30 °C reached about 89, 103 and 221, respectively. This showed that the nanosheet membrane gave excellent molecular sieve performance for H_2_ over other gases, especially for H_2_ over CO_2_, demonstrating the ultrahigh separation function of the nanosheet membrane for small-molecule mixtures. In addition, to further assess the mechanical stability of the obtained membrane, the permeation performances of gas molecules were measured at different trans-membrane pressure drops as shown in Fig. S9.† The results show that both gas permeances and H_2_/CO_2_ separation selectivity through the nanosheet membrane could almost remain unchanged when the H_2_ partial pressure increased from 0.5 to 1.5 bar, thus demonstrating excellent mechanical stability. The permeances of all the gases through the membrane are independent of the trans-membrane pressure drop. This implies that the prepared nanosheet membrane is compact and contains no pinholes or defects.

The influence of temperature on the gas permeance and selectivity of H_2_/CO_2_ and the long-term permeation performances of gas molecules for the achieved nanosheet tubular membrane were also investigated to evaluate its thermal and operating stability as shown in Fig. 8b and c. The permeation behaviors of both H_2_ and CO_2_ hardly changed within the temperature range from 30 to 150 °C. However, both the permeance of H_2_ and separation selectivity of H_2_/CO_2_ slightly increased at elevated temperature as shown in Fig. 8b. This is probably due to the structural flexibility of the nanosheets, which brings about a slight increase in the effective pore size of the nanosheet membrane with increasing the temperature. As the kinetic diameter of CO_2_ is larger than the aperture size of the nanosheets, this tiny change of pore size can cause a slight increase in the permeance of H_2_ and hardly has an influence on the permeance of CO_2_, thus resulting in a slight increase in the separation selectivity of H_2_/CO_2_ at elevated temperature.^19^

Fig. 8c shows the long-term operating performances of gas molecules through the M-9 nanosheet membrane measured from 30 to 150 °C at 0.1 MPa. The M-9 membrane was firstly tested at 30 °C for 100 h for investigating the transport property of the H_2_/CO_2_ binary mixture through the membrane. Then, the membrane was heated to 100 °C and exposed to equimolar H_2_/CO_2_ binary gas mixtures for 100 h. After that, the membrane was heated up to 150 °C and tested for 100 h before cooling down to room temperature. It is seen that there are barely changes for the permeances of both H_2_ and CO_2_ during the operating period of 400 h within the temperature range from 30 to 150 °C. No degradation in membrane performance was observed within the entire test except that there was a slight increase in the gas selectivity of H_2_/CO_2_ with elevated temperature because of a tiny increase of H_2_ permeance. Hence, this long-term permeation result showed that the nanosheet tubular membrane has excellent thermal and operating stability.

Here a comparison of our achieved Zn_2_(bIm)_4_ nanosheet *tubular* membrane with some membranes reported in previous references for H_2_/CO_2_ separation was also made. As shown in Table S1† and Fig. 8d in which the black solid line represents the 2008 upper bound of polymeric membranes for H_2_/CO_2_, and the black dashed line represents the 2010 upper bound of microporous inorganic membranes for the separation of H_2_/CO_2_ mixtures, our prepared nanosheet membrane far surpassed the trade-off line and had much better separation performance for H_2_/CO_2_ than most of the other reported microporous molecular sieve membranes including zeolites, MOFs and polymers, demonstrating that our highly oriented nanosheet membrane is of high quality and has excellent gas separation performance for H_2_/CO_2_ mixtures. This suggests that highly oriented nanosheets play an important role in transporting gases through the molecular sieve membranes. Compared to the Zn_2_(bIm)_4_ nanosheet membrane with around 5 nm thickness supported on a porous disc by the exfoliation–deposition method reported by Yang's group,^14^,^19^ indeed, our membrane gave a lower performance for H_2_/CO_2_. The differences may mainly result from the substrate difference for membrane preparation. It is well recognized that the substrate quality seriously influences the membrane quality and its separation performance.^5^,^44^–^46^ Our nanosheet membrane obtained here was grown on a porous tubular substrate with a larger pore size, coarser surface and larger area. Compared to a porous disc with both smaller pores and smaller area, this porous tubular tube usually contains more defects including macropores, needle holes and cracks as shown in Fig. S10,† thus easily producing some membrane defects for growing a thinner nanosheet membrane. Of course, further optimization and improvement for our present method are still needed. Because the ZnO NPs used here are very small with size no more than 50 nm, an uncontinuous ZnO NP layer is easily formed by coating on the tube. So, the nanosheet membrane has to grow on the thicker ZnO layer coated on the tube and form a thicker membrane so as to avoid the defects of the membrane. In fact, thinner nanosheet ZIF membranes could exhibit better H_2_/CO_2_ gas separation performance.^19^,^25^ Moreover, it is much more challenging to fabricate a defect-free and thin crystalline nanosheet membrane on a *tubular* substrate than a *planar* one. Unfortunately, by this strategy, we have not achieved a much thinner nanosheet membrane yet by now. Nevertheless, the separation performance of our nanosheet membrane is still much higher than those of other molecular sieve membranes reported to date (Fig. 8d and Table S1†). More importantly, our membranes were fabricated on large-scale tubular substrates which are more feasible in industrial applications.

To verify the versatility of our novel preparation strategy, 2D Zn-based MOF materials, ZnBDC (BDC = phthalic acid) and ZnBTC (BTC = 1,3,5-benzenetricarboxylic acid), were chosen to prepare their corresponding nanosheet membranes as shown in Fig. S11.† From the SEM images, the supports were covered with highly oriented nanosheet membranes and no visible pinholes or other defects were observed. Evidenced by the XRD patterns, the as-prepared nanosheets are typical ZnBDC and ZnBTC structures, respectively, and the membranes are highly oriented. Tables S2 and S3† show the permeances of single gases through the ZnBDC and ZnBTC membranes as a function of the kinetic diameter of the permeating molecules at 30 °C. The results show that both the nanosheet membranes demonstrate excellent molecular sieve performances for H_2_ over other gases. Therefore, this bottom-up synthesis approach can be extended to prepare other kinds of Zn-based MOF nanosheet membranes through self-conversion of zinc oxide@GO in a GO-confined space on a porous support. Further study is currently being pursued in our lab.

## 4. Conclusions

In conclusion, we for the first time developed a direct growth strategy (*i.e.* bottom-up method) to prepare a highly oriented ZIF nanosheet *tubular* membrane. Excellent gas separation performance with a H_2_ permeance of 1.5 × 10^–7^ mol m^–2^ s^–1^ Pa^–1^ and a H_2_/CO_2_ ideal separation selectivity of 106 was achieved through our membrane. The method is based on self-conversion of a thin layer of ZnO NPs confined between the surface of a tubular substrate and an ultrathin layer of GO into a nanosheet membrane. The ZnO NP layer acts as seeds and anchoring sites for the membrane growth, while the GO layer on the top of the ZnO NP layer can confine and guide the oriented growth of nanosheets due to the interaction of the abundant carboxylate groups at the surfaces and edges of the GO layer with the Zn_2_(bIm)_4_ nanosheets generated at the initial stage. The highly oriented nanosheet membrane achieved is like a window-frame structure exhibiting excellent operating stability. Moreover, this method can also be used for the synthesis of other Zn-based MOF nanosheet membranes such as ZnBDC and ZnBTC. It is believed that this work opens up a simple and scalable direct growth route for achieving a highly oriented nanosheet MOF membrane, especially a *tubular* membrane for potential industrial applications.

## Conflicts of interest

There are no conflicts to declare.

## Supplementary Material

Supplementary information
