# Supplementary material for: GO-guided direct growth of highly oriented metal–organic framework nanosheet membranes for H2/CO2 separation
Source: Chem Sci. 2018 Apr 2;9(17):4132–41. doi: 10.1039/c7sc04815g (PMC5941280; doi:10.1039/c7sc04815g)
Supplement: Supplementary file 1 [file SC-009-C7SC04815G-s001.pdf]

Electronic Supplementary Material (ESI) for Chemical Science.
This journal is © The Royal Society of Chemistry 2018

# Electronic Supplementary Material

# GO-guided direct growth of highly oriented metal-organic framework nanosheet membranes for $H_2/CO_2$ separation

*Yujia Li[1], Haiou, Liu[1], Huanting Wang[2] , JieshanQiu[1] , Xiongfu Zhang[1] **

[1]State Key Laboratory of Fine Chemicals, School of Chemical Engineering,
Dalian University of Technology, Dalian, 116024, China
**Corresponding author*: xfzhang@dlut.edu.cn (X. Zhang).

[2]Department of Chemical Engineering, Monash University,
Clayton, Victoria 3800, Australia

## Experimental section

### Preparation of GO

GO suspension was prepared from natural graphite powder using the modified Hummers method as reported in the literature. Graphite powder (325 meshes, 1.25 g) was mixed in sulfuric acid (30 mL, 98 wt%) in an ice bath under stirring for 5 min before adding 0.625 g of sodium nitrate ($NaNO_3$). After stirring for 2 h, 5.62 g of $KMnO_4$ (5.62 g) was slowly added into the suspension at a temperature below 20 $^oC$ and the mixture was sequentially stirred for 2 h. Afterwards, the mixture suspension was transferred to a 35 $^oC$ water bath for 30 min to form a thick paste. After diluting with 58 mL of deionized water, the suspension was stirred for 30 min at 98 $^oC$. Then the suspension was treated with 1.75 mL of $H_2O_2$ (30 wt%) to tune its color to yellow after the addition of additional 140 mL of 60 $^oC$ water. The suspension was centrifuged and dialysis in water to wash. GO suspension was obtained.

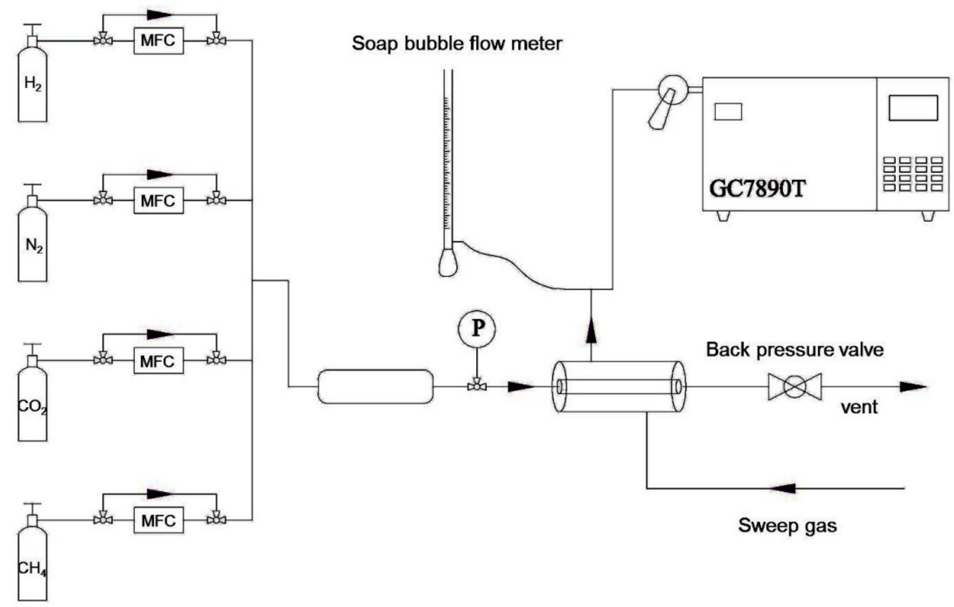


**Figure S1.** Schematic diagram of the gas permeation apparatus.

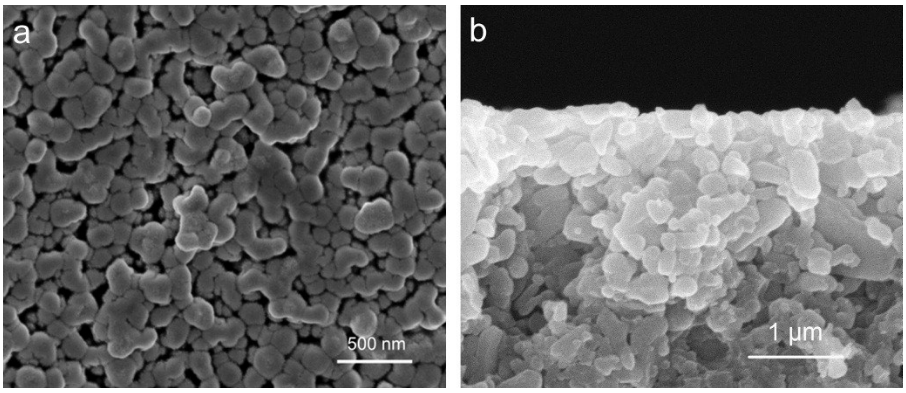


**Figure S2.** SEM images of the porous α-$Al_2O_3$ tube: Top view; Cross-section view

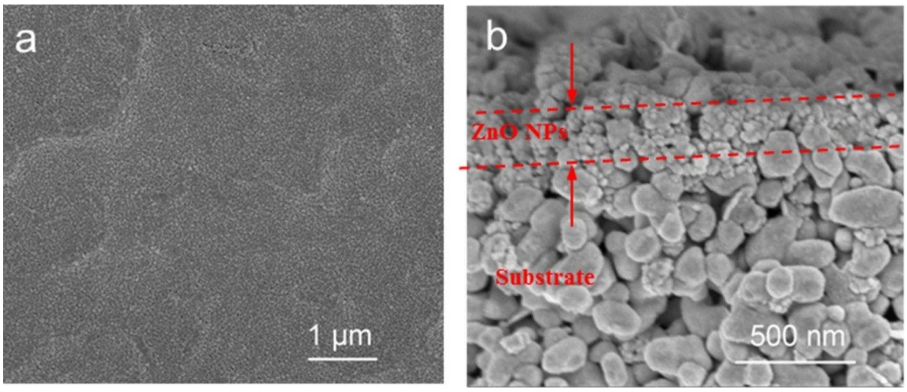


**Figure S3.** SEM images of the tube coated with ZnO NPs: Top view and Cross-section view.

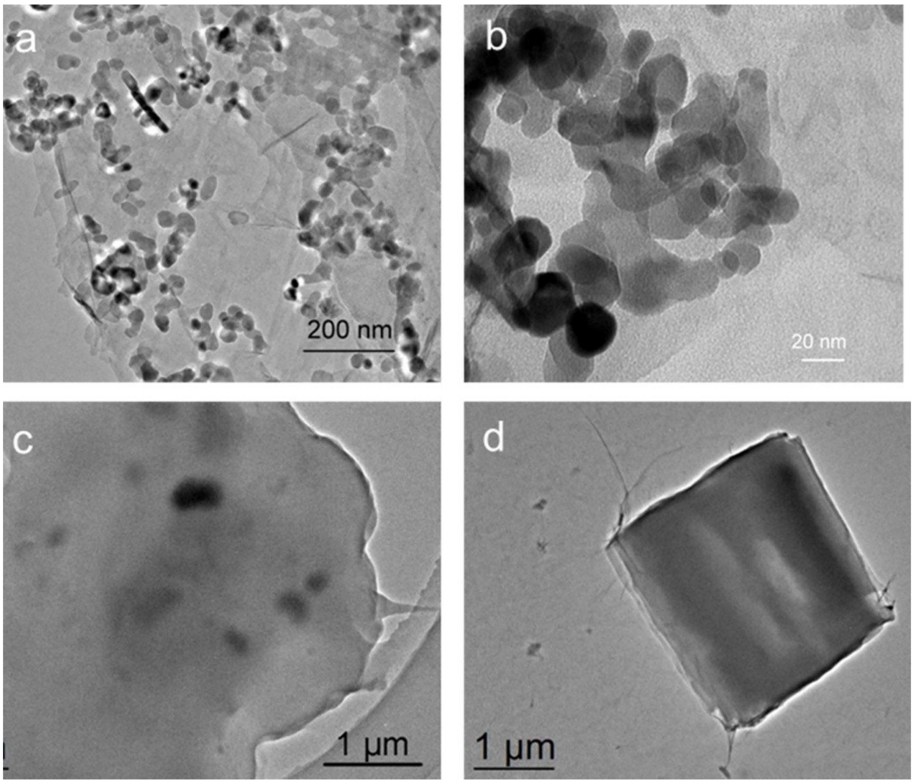


**Figure. S4** TEM images of ZnO@GO nanocomposite (a, b) and the samples (c, d) achieved by self conversion of ZnO nanoparticles @ GO in the synthesis solution after reaction for 1 h and 5 h, respectively.

As shown in Figure S4, the extent of the conversion of ZnO@GO was investigated using bright-field transmission electron microscopy (BR-TEM). In the early stages of the reaction, the surfaces of the ZnO NPs are coated with GO (Figure S4 a,b). With prolonging reaction time (1 h), the nanosheet precursors grow and form continuous layers (Figure S4 c). As the reaction proceeds, the nanosheets become larger, while the ZnO NPs become smaller, until the NPs are completely converted (Figure S4 d). These results suggest that the nanosheets were mainly formed through the localized conversion of ZnO NPs, rather than by dissolution of ZnO and following crystallization.

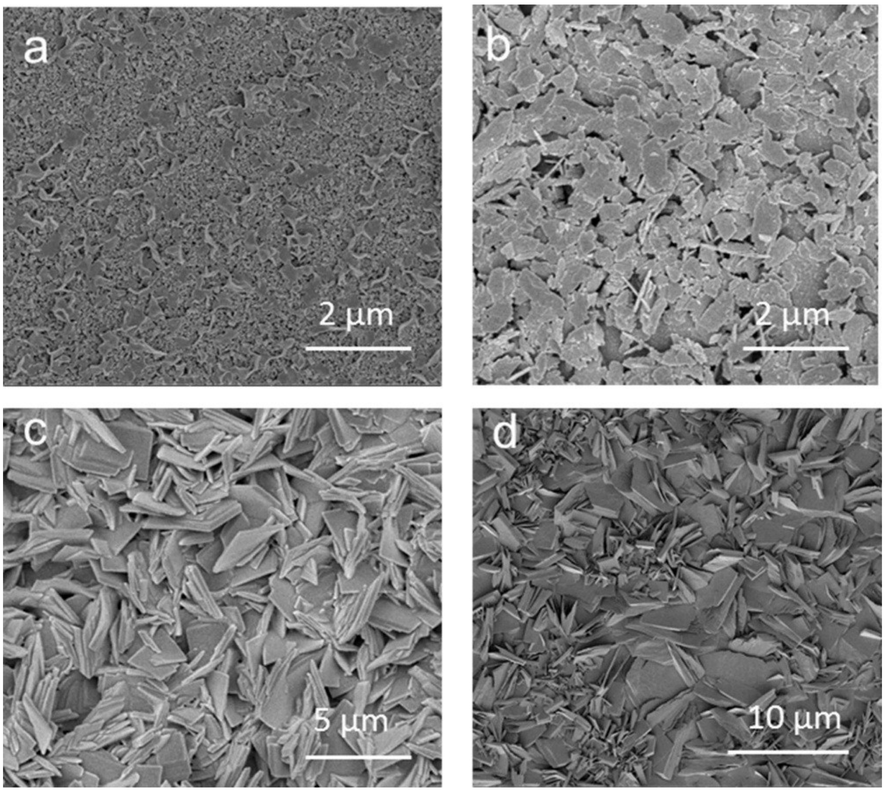


**Figure S5.** SEM images of the samples obtained by self-conversion of ZnO NPs without GO layer for the reaction of 1 h (a), 3 h (b), 7 h (c) and 24 h (d).

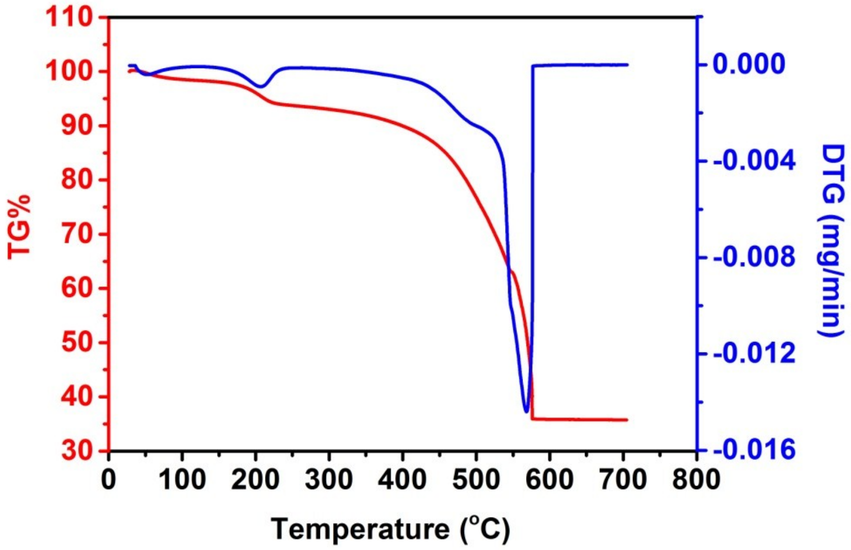


**Figure. S6** TG curves of $Zn_2(bim)_4$ nanosheets.

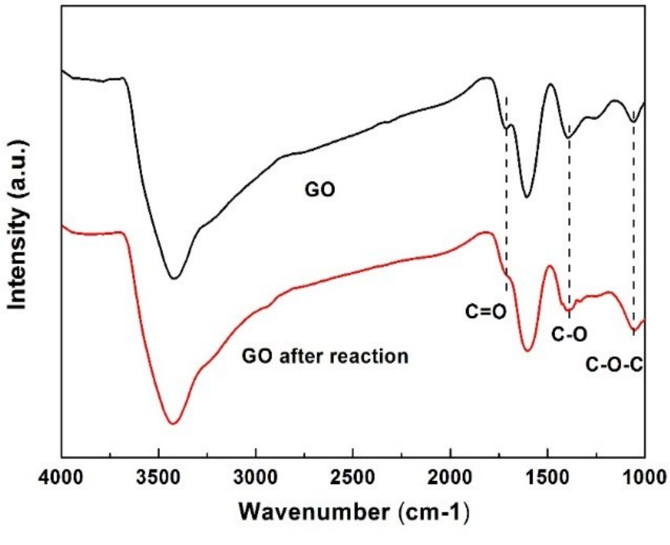


**Fig. S7** FTIR spectra of the GO before and after the reaction with the same conditions as the membrane synthesis

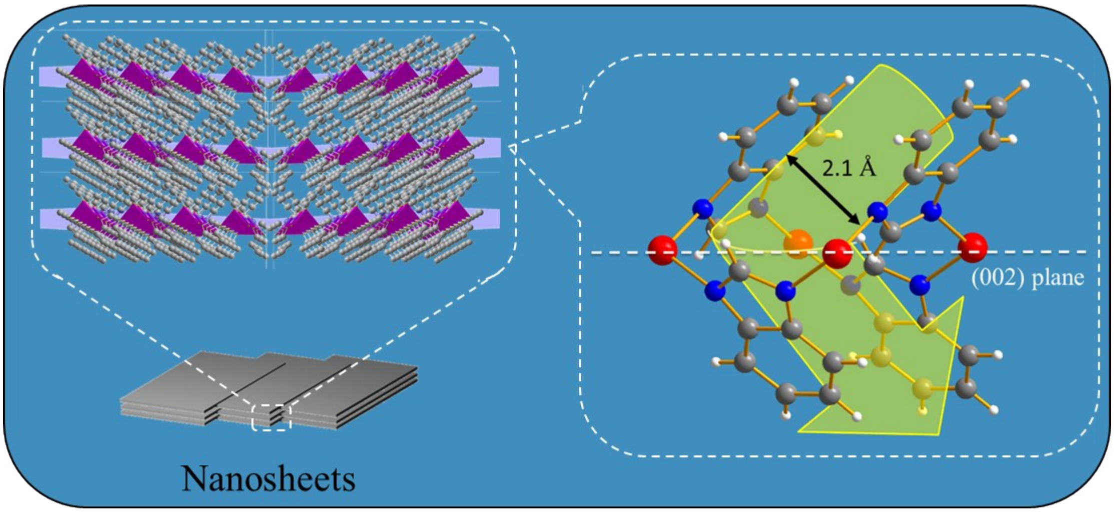


**Figure. S8** 3D crystalline structure along *c* crystallographic axes showing the stacking direction and pore system of the ZIF nanosheet. Zn, N, C, and H atoms are shown in red, blue, gray, and white, respectively.

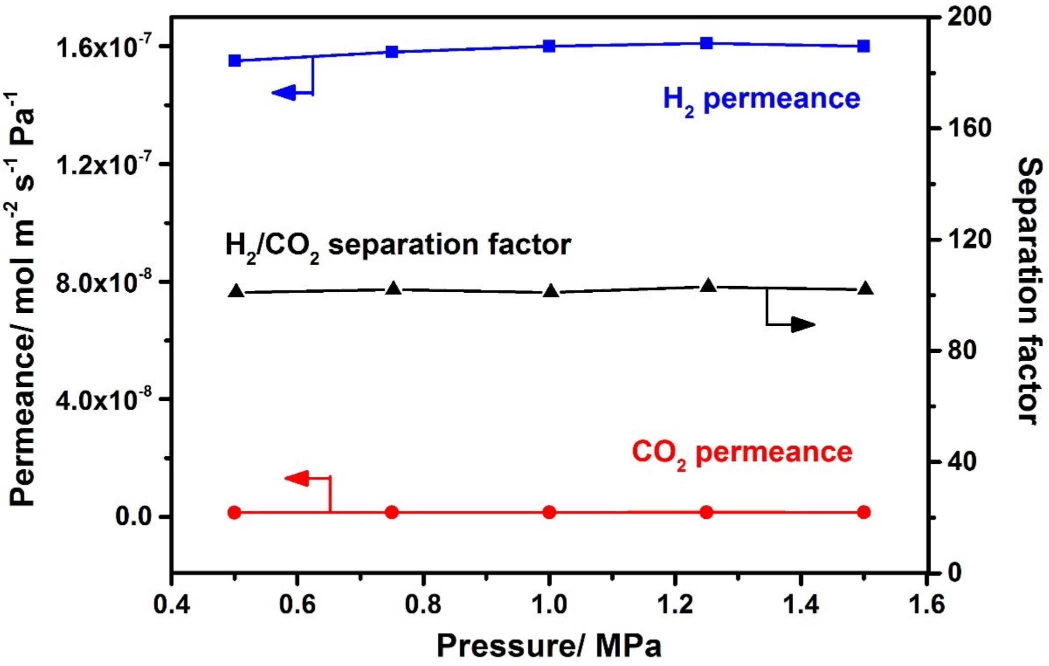


**Figure S9**. $H_2/CO_2$ selectivity and permeances of the ZIF nanosheet membrane prepared on porous α-alumina tubes as function of the $H_2$ partial pressure at 30 °C.

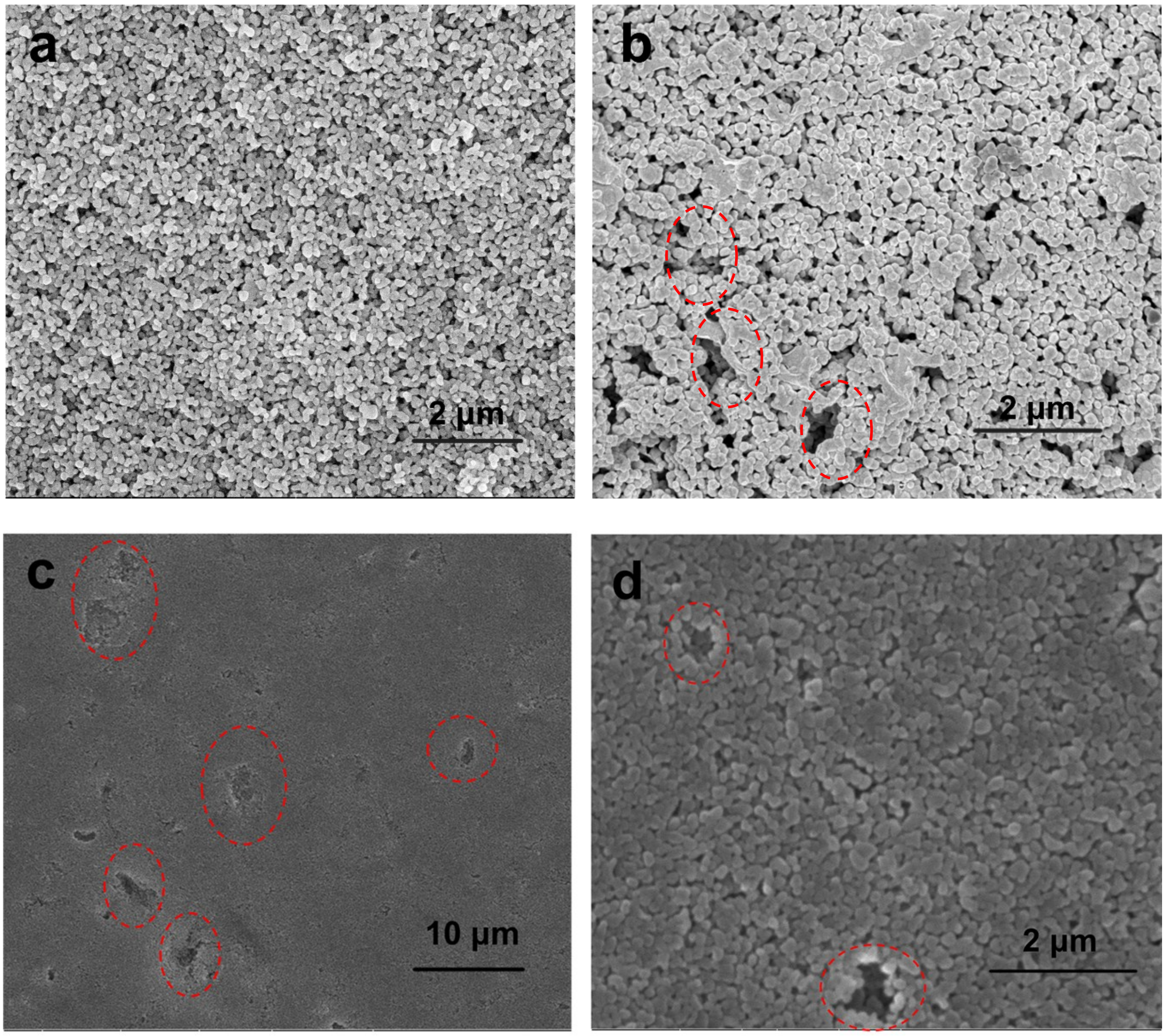


Figure S10. SEM images of the bare surface of the two substrates and the surface of the tube coated with ZnO NPs

(a) Ceramic disk of 70 nm used by Yang;

(b and c) Ceramic tube of 100 nm used in our study;

(d) Surface of the ceramic tube coated with ZnO NPs

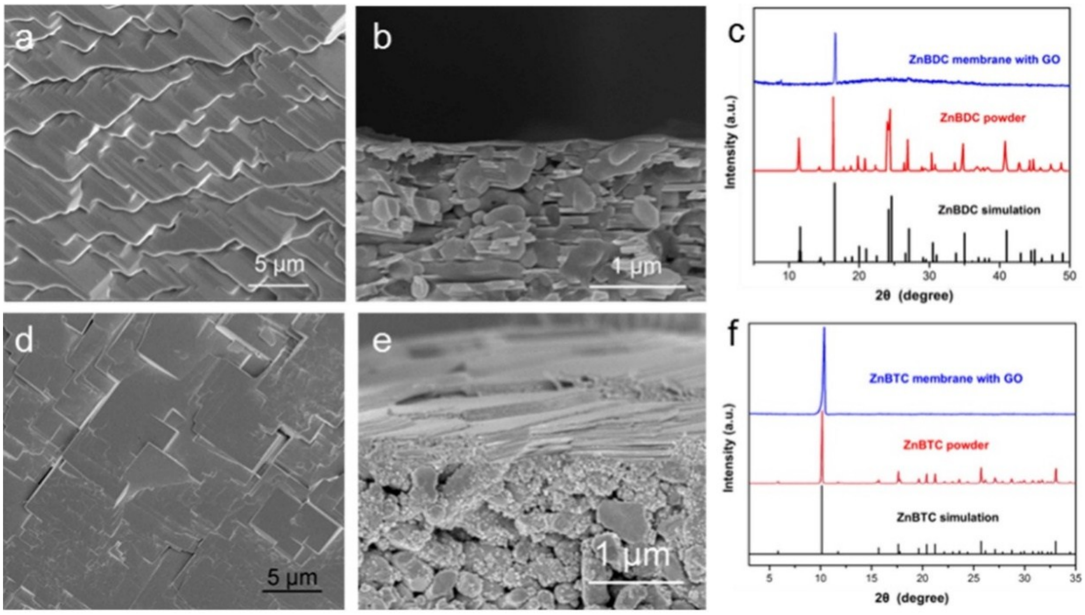


**Figure. S11**. SEM images of (a, b and c) ZnBDC (BDC: p-phthalic acid) and (d, e and f) ZnBTC (BTC: 1,3,5-benzenetricarboxylic acid) nanosheet membranes formed with ZnO self-conversion strategy with the guide of GO. XRD patterns of the membranes grown with GO and without GO, respectively, and the simulated pattern of ZnBDC and ZnBTC

ZnBDC is a kind of microporous framework material consisting of Zinc and BDC ligand [1], while ZnBTC, composed of Zn and BTC ligand, is an undulating 2-D network [2], with individually chiral, pseudo trigonal pores constructed rom windmill- or propeller-like building blocks.

Similar to the synthesis conditions of the $Zn_2(bIm)_4$ nanosheet membrane, we used the synthesis solution with p-phthalic acid (BDC) or 1,3,5-benzenetricarboxylic acid (BTC) as ligand and DMF as solvent to prepare different samples as shown in Figure S6. The ZnBDC and ZnBTC powders were formed and all the nanosheets randomly. XRD pattern (Figure S6 c and f) clearly shows that there is presence of all peaks for ZnBDC and ZnBTC structure, indicating the powders are not an oriented one. However, with the aid of the GO layer on the top of the ZnO nanoparticles layer, two kinds of highly oriented ZnBDC and ZnBTC nanosheet membranes were successfully achieved, indicating excellently guiding role of GO.

**References**

1. H. Li, M. Eddaoudi, T. L. Groy, O. M. Yaghi, *J. Am. Chem. Soc.*, **1998**, 120, 8571.
2. G. Wu, X. Shi, Q. Fang, G. Tian, L. Wang, G. Zhu, A. W. Addison, Y. Wei, S. Qiu, *Chem. Commun.*, **2003**, 6, 402.

**Table S1. Comparison between the MOF membranes reported by literatures and this work**

| Membrane | Substrate | Temperature (°C) | Selectivity ($H_2/CO_2$) | Permeance (GPU) | Transmembrane pressure (bar) | Ref |
|---|---|---|---|---|---|---|
| $Zn_2(bIm)_4$ MSN | Disk | 120 | 291 | 2300 | 0 | 1 |
| CuBTC/MIL-100 | Tube | 85 | 89 | 300 | 1 | 2 |
| $NH_2$-MIL-53(Al) | Disk | 15 | 31 | 5050 | 0 | 3 |
| ZIF-95 | Disk | 120 | 25.7 | 5890 | 0 | 4 |
| ZIF-8/ZIF-9 | Tube | 150 | 9.6 | 250 | 1 | 5 |
| ZIF-8@GO | Disk | 250 | 14.9 | 570 | 0 | 6 |
| ZIF-9 | Disk | 25 | 14.7 | 2190 | 1 | 7 |
| ZIF-7 | Disk | 220 | 13.6 | 116 | 0 | 8 |
| COF-300/ZIF-8 | Disk | 25 | 13.5 | 11 | 1 | 9 |
| CAU-1 | Disk | 25 | 13.3 | 491 | 0 | 10 |
| NiAl-$CO_3$ LDH | Disk | 180 | 10.8 | 43.3 | 0 | 11 |
| Polyimide/ZIF-11 | Disk | 200 | 9.1 | 535 | 1.7 | 12 |
| ZIF-22 | Disk | 50 | 7.2 | 521 | 0 | 13 |
| ZIF-8 | Tube | 25 | 5.2 | 600 | 0 | 14 |
| GO | Disk | 20 | 3400 | 900 | 0 | 15 |
| ZIFs/rGO | Disk | 170 | 26.4 | 151 | 0 | 16 |
| MAMS-1 | Disk | 25 | 34 | 6516 | 0 | 17 |
| Nanosheet ZIF membrane | Tube | 150 | 106 | 415 | 0 | This work |

**References**


1. Y. Peng, Y. Li, Y. Ban, H. Jin, W. Jiao, X. Liu and W. Yang, *Science,* 2014, **346**, 1356.

2. W. Li, Y. Zhang, C. Zhang, Q. Meng, Z. Xu, P. Su, Q. Li, C. Shen, Z. Fan, L. Qin, G. Zhang, *Nat. Commun.* **2016**, 7, 11315.

3. F. Zhang, X. Zou, X. Gao, S. Fan, F. Sun, H. Ren, G. Zhu, *Adv. Funct. Mater*. **2012**, 22, 3583.

4. A. Huang, Y. Chen, N. Wang, Z. Hu, J. Jiang, J. Caro, *Chem. Commun.* **2012**, 48, 10981.

5. F. Cacho-Bailo, I. Matito-Martos, J. Perez-Carbajo, M. Etxeberria-Benavides, O. Karvan, V. Sebastian, S. Calero, C. Tellez, J. Coronas, *Chem. Sci.* **2017**, 8, 325.

6. A. Huang, Q. Liu, N. Wang, Y. Zhu, J. Caro, *J. Am. Chem. Soc.* **2014**, 136, 14686.

7. Y. Huang, D. Liu, Z. Liu, C. Zhong, *Ind. Eng. Chem. Res.* **2016**, 55, 7164.

8. Y. Li, F. Liang, H. Bux, W. Yang, J. Caro, *J. Membr. Sci.* **2010**, 354, 48.

9. J. Fu, S. Das, G. Xing, T. Ben, V. Valtchev, S. Qiu, *J. Am. Chem. Soc.* **2016**, 138, 7673.

10. S. Zhou, X. Zou, F. Sun, H. Ren, J. Liu, F. Zhang, N. Zhao, G. Zhu, *Int. J. Hydrogen. Energ.* **2013**, 38, 5338.

11. Y. Liu, N. Wang, J. H. Pan, F. Steinbach, J. Caro, *J. Am. Chem. Soc.* **2014**, 136, 14353.

12. J. Sánchez-Laínez, B. Zornoza, Á. Mayoral, Á. Berenguer-Murcia, D. Cazorla-Amorós, C. Téllez, J. Coronas, *J. Mater. Chem. A* **2015**, 3, 6549-6556.

13. A. Huang, H. Bux, F. Steinbach, J. Caro, *Angew. Chem. Int. Ed.* **2010**, 49, 4958.

14. X. Zhang, Y. Liu, L. Kong, H. Liu, J. Qiu, W. Han, L. Weng, K. L. Yeung and W. Zhu, *J. Mater. Chem. A,* 2013, **1**, 10635.

15. H. Li, Z. N. Song, X. J. Zhang, Y. Huang, S. G. Li, Y. T. Mao, H. J. Ploehn, Y. Bao, M. Yu, *Science* **2013**, 342, 95.

16. W. Li, Y. Zhang, P. Su, Z. Xu, G. Zhang, C. Shen, Q. Meng, *J. Mater. Chem. A* **2016**, 4, 18747.

17. X. Wang, C. Chi, K. Zhang, Y. Qian, K. M. Gupta, Z. Kang, J. Jiang, D. Zhao, *Nat. Commun.* **2017**, 8, 14460.

**Table S2** Single gas separation properties through the ZnBDC membrane at 303 K.

| Gas i/j | Knudsen constant | Performances of ZnBDC membrane | | |
|---|---|---|---|---|
| | | Single gas/ P $10^{-9}$ mol·m$^{-2}$·s$^{-1}$·Pa$^{-1}$ | | |
| | | ( i ) | ( j ) | I. S. |
| $H_2/CO_2$ | 4.7 | 12.31 | 0.78 | 15.8 |
| $H_2/N_2$ | 3.7 | 12.31 | 0.37 | 33.1 |
| $H_2/CH_4$ | 2.8 | 12.31 | 0.22 | 56.6 |

**Table S3** Single gas separation properties through the ZnBTC membrane at 303 K.

| Gas i/j | Knudsen constant | Performances of ZnBTC membrane | | |
|---|---|---|---|---|
| | | Single gas/ P $10^{-9}$ mol·m$^{-2}$·s$^{-1}$·Pa$^{-1}$ | | |
| | | ( i ) | ( j ) | I. S. |
| $H_2/CO_2$ | 4.7 | 9.78 | 0.41 | 24.1 |
| $H_2/N_2$ | 3.7 | 9.78 | 0.17 | 57.1 |
| $H_2/CH_4$ | 2.8 | 9.78 | 0.10 | 98.6 |

P: Permeances; I.S.: Ideal separation factor
